# A multi-modal deep learning approach for stress detection using physiological signals: integrating time and frequency domain features

**DOI:** 10.3389/fphys.2025.1584299

**Published:** 2025-04-01

**Authors:** Jun-Zhi Xiang, Qin-Yong Wang, Zhi-Bin Fang, James A. Esquivel, Zhi-Xian Su

**Affiliations:** ^1^ Emergency Department, The First Affiliated Hospital of Wenzhou Medical University, Wenzhou, Zhejiang, China; ^2^ School of Artificial Intelligence, Zhejiang College of Security Technology, Wenzhou, Zhejiang, China; ^3^ School of Software Technology, Zhejiang University, Ningbo, Zhejiang, China; ^4^ Graduate School, Angeles University Foundation, Angeles, Philippines

**Keywords:** multi-modal deep learning, time and frequency domain features, fast fourier transform, stress detection, wearable devices

## Abstract

**Objective:**

This study aims to develop a multimodal deep learning-based stress detection method (MMFD-SD) using intermittently collected physiological signals from wearable devices, including accelerometer data, electrodermal activity (EDA), heart rate (HR), and skin temperature. Given the unique demands and high-intensity work environment of the nursing profession, stress measurement in nurses serves as a representative case, reflecting stress levels in other high-pressure occupations.

**Methods:**

We propose a multimodal deep learning framework that integrates time-domain and frequency-domain features for stress detection. To enhance model robustness and generalization, data augmentation techniques such as sliding window and jittering are applied. Feature extraction includes statistical features derived from raw time-domain signals and frequency-domain features obtained via Fast Fourier Transform (FFT). A customized deep learning architecture employs convolutional neural networks (CNNs) to process time-domain and frequency-domain features separately, followed by fully connected layers for final classification. To address class imbalance, the Synthetic Minority Over-sampling Technique (SMOTE) is utilized. The model is trained and evaluated on a multimodal physiological signal dataset with stress level labels.

**Results:**

Experimental results demonstrate that the MMFD-SD method achieves outstanding performance in stress detection, with an accuracy of 91.00% and an F1-score of 0.91. Compared to traditional machine learning classifiers such as logistic regression, random forest, and XGBoost, the proposed method significantly improves both accuracy and robustness. Ablation studies reveal that the integration of time-domain and frequency-domain features plays a crucial role in enhancing model performance. Additionally, sensitivity analysis confirms the model’s stability and adaptability across different hyperparameter settings.

**Conclusion:**

The proposed MMFD-SD model provides an accurate and robust stress detection approach by integrating time-domain and frequency-domain features. Designed for occupational environments with intermittent data collection, it effectively addresses real-world stress monitoring challenges. Future research can explore the fusion of additional modalities, real-time stress detection, and improvements in model generalization to enhance its practical applicability.

## 1 Introduction

In the fast-paced modern world, stress has emerged as a pervasive and significant health concern, affecting individuals across all walks of life. The World Health Organization has declared stress a global epidemic, with its impacts ranging from decreased productivity and quality of life to severe physical and mental health issues. As awareness of these detrimental effects grows, so does the need for accurate, real-time stress detection methods that can facilitate timely interventions and support effective stress management strategies.

Traditional approaches to stress assessment have largely relied on self-reports and occasional clinical evaluations. However, these methods are limited by their subjective nature, infrequency, and inability to capture real-time stress fluctuations. The advent of wearable technology has opened new avenues for continuous, objective stress monitoring through the measurement of various physiological signals (Ceren Ates et al., 2024; Mozos et al., 2016). These devices can capture a wealth of data, including heart rate variability, electrodermal activity, skin temperature, and accelerometer data, providing a more comprehensive picture of an individual’s physiological state.

Despite this technological advancement, the challenge of accurately interpreting these multi-modal physiological signals to detect stress remains significant. Early attempts at physiological stress detection often focused on single-modal approaches, utilizing individual biomarkers such as heart rate or skin conductance. While these methods showed promise, they failed to capture the complex, multi-faceted nature of the human stress response (Gedam and Paul, 2021; Delmastro et al., 2020). More recent studies have explored multi-modal approaches, combining data from various physiological signals to improve detection accuracy. However, many of these methods still rely heavily on time-domain features, potentially overlooking valuable information contained in the frequency domain of these signals (Zhao et al., 2024).

The integration of machine learning techniques, particularly deep learning, has shown great potential in improving stress detection accuracy (Rogerson et al., 2023). CNNs and Recurrent Neural Networks (RNNs) have been successfully applied to time-series physiological data, demonstrating their ability to capture complex patterns and relationships. However, the majority of these approaches still primarily focus on time-domain features, leaving the rich information in the frequency domain largely unexplored.

An important consideration in stress detection research, particularly in occupational settings such as healthcare, is the intermittent nature of data collection. Wearable sensors typically collect data during work hours but not during rest periods. This intermittent data collection reflects the reality of work-life balance and presents both challenges and opportunities for stress detection algorithms (Kyriakou et al., 2019). On one hand, it necessitates robust methods that can handle gaps in data collection. On the other hand, it focuses the analysis on periods when occupational stress is most likely to occur, potentially increasing the relevance of the collected data (Smets et al., 2018).

In this context, the nursing profession serves as a particularly illustrative example of occupational stress measurement. Due to the unique demands, high work intensity, and often challenging environments nurses face, their stress measurement is not only significant but also representative of stress levels in other high-pressure occupations. Thus, this study specifically targets stress detection in nurses as a focal point for research.

To address these challenges, this study proposes a novel multi-modal deep learning approach for stress detection using physiological signals. The method integrates both time and frequency domain features extracted from various physiological signals, including accelerometer data (X, Y, Z), EDA, HR, and TEMP (Ceren Ates et al., 2024). By combining these diverse data sources and feature types, the aim is to capture a more comprehensive representation of the stress response.

The approach leverages advanced signal processing techniques, including FFT, to extract rich spectral features from the physiological signals. These frequency-domain features are then combined with traditional time-domain features to provide a holistic view of the physiological data. To process this multi-modal data effectively, a custom deep learning architecture is designed that employs parallel CNNs to separately handle the time-domain and frequency-domain features before merging them for final classification.

Specifically, the method involves several key steps. First, the raw physiological signals are preprocessed to remove noise. Then, a comprehensive set of time-domain features is extracted, including statistical measures such as mean, standard deviation, and percentiles, as well as physiological-specific features like heart rate variability measures. In parallel, FFT is applied to the signals to obtain their frequency-domain representations, from which spectral features such as power in different frequency bands are extracted.

These extracted features are then fed into the custom CNNs architecture. The architecture consists of two parallel CNNs branches: one for time-domain features and another for frequency-domain features. Each branch contains multiple convolutional layers followed by pooling layers to learn hierarchical representations of the features. The outputs of these parallel branches are then concatenated and passed through fully connected layers for final stress classification.

The main contributions of this work are summarized as follows:

### 1.1 Multi-modal integration architecture

Our approach uniquely combines both time and frequency domain features from multiple physiological signals (accelerometer, EDA, HR, and TEMP) through a novel parallel CNN architecture. This dual-domain processing strategy allows for capturing complementary stress manifestations that might be missed in traditional single-domain approaches.

### 1.2 Innovative feature processing

While individual components like FFT and CNN are established techniques, our implementation innovatively combines them in parallel pathways to process different feature types. This architectural design enables simultaneous analysis of both instantaneous physiological responses (time-domain) and rhythmic patterns (frequency-domain) in stress manifestations.

### 1.3 Adaptation for intermittent data

Our methodology specifically addresses the challenges of intermittent data collection in occupational settings - a crucial real-world constraint often overlooked in conventional approaches. The model’s design accommodates the discontinuous nature of workplace physiological monitoring through specialized data segmentation and augmentation techniques.

### 1.4 Custom deep learning architecture

The proposed architecture is specifically designed for stress detection, featuring parallel CNNs that process time and frequency domain features independently before merger. This design differs from conventional approaches by allowing each domain to be processed optimally before integration.

### 1.5 Comprehensive signal integration

Our method uniquely integrates multiple physiological signals while maintaining their individual characteristics through separate processing pathways, rather than simple concatenation used in conventional approaches.

This comprehensive set of contributions positions this work as a significant advancement in the field of physiological stress detection, offering a more accurate and adaptable approach to this critical health monitoring task. The multi-modal deep learning method addresses key challenges in the field and opens new avenues for research in stress detection and overall wellbeing enhancement, particularly in occupational settings where continuous monitoring is not feasible or practical.

## 2 Related work

Wearable devices coupled with machine learning techniques have emerged as powerful tools for stress detection, offering continuous, non-invasive monitoring capabilities in real-world environments. A comprehensive review highlighted the significance of physiological indicators, including heart rate variability (HRV), skin temperature, and EDA in stress detection (Gedam and Paul, 2021). This work emphasized the crucial role of both time-domain and frequency-domain analyses for precise stress monitoring. However, existing studies often focus on either time-domain or frequency-domain features separately, limiting their ability to fully capture stress-related physiological variations. Subsequently, a systematic review presented generalizable machine learning models for stress monitoring, addressing critical challenges such as dataset transferability and model robustness across diverse populations (Vos et al., 2023). While these models improve generalizability, they often overlook the challenges posed by intermittent data collection in real-world occupational settings.

Recent advances in predictive modeling have demonstrated the effectiveness of integrating multiple data sources. Comparative studies examining various stress prediction models that combine smartwatch physiological signals with self-reported measures revealed enhanced predictive performance through this dual-source approach (Dai et al., 2021). Nevertheless, reliance on self-reported data introduces subjectivity, which may affect model reliability and applicability in real-time monitoring. In parallel, research introduced an explainable deep learning framework for stress detection using wearable sensor data, providing crucial transparency in model interpretation for healthcare applications (Moser et al., 2024). Although explainability improves trust in deep learning models, further enhancements are needed to balance interpretability with predictive accuracy. Furthermore, investigations into autoencoder-based approaches demonstrated the effectiveness of temporal feature extraction from wearables for forecasting both stress and mood, highlighting the potential of unsupervised learning methods in personalized health monitoring (Li and Sano, 2020). Despite their success, autoencoder-based methods often require extensive tuning and may struggle with diverse physiological patterns in occupational stress scenarios.

Recent sensor-based methods have advanced stress detection by integrating new data modalities. For example, magnetostrictive polymer composites (MPCs) using UV-curable epoxy resin demonstrated reliable stress detection through changes in magnetic flux, offering potential to refine stress monitoring systems by augmenting time and frequency domain features (Paul et al., 2024). While this approach showcases novel sensor technology, its practicality for widespread wearable integration remains uncertain.

Furthermore, deep learning advancements in sensor-based recognition have enabled automatic feature extraction across complex physiological signals, addressing challenges such as unsupervised and incremental learning. These frameworks improve adaptability and interpretability, enhancing stress detection in varied real-world contexts (Wang et al., 2019). However, many existing models lack mechanisms to effectively integrate multi-modal data, limiting their ability to capture stress responses comprehensively.

The role of specific physiological parameters in stress detection has been extensively investigated. Novel methods for mental stress assessment using HRV derived from electrocardiogram (ECG) signals demonstrated high precision in stress quantification (Saini and Gupta, 2024). Despite their accuracy, ECG-based approaches often require specialized sensors, reducing feasibility for daily wear. Additionally, pilot studies contributed to the field through the introduction of the Stress-Predict dataset, establishing a robust foundation for developing and validating stress prediction algorithms across diverse conditions (Iqbal et al., 2022). While valuable for benchmarking, these datasets may not fully represent stress variability in high-intensity professional settings. Research into the feasibility of combining wearable and self-reported measures in controlled lab environments has illuminated both the potential and limitations of deploying these techniques in real-world applications (Aristizabal et al., 2021). Yet, stress assessment in controlled environments may not directly translate to occupational settings where intermittent data collection is a major challenge.

In professional environments, research explored embedded devices for continuous stress monitoring, providing valuable insights into wearable adaptation for demanding workplace settings (Kafková et al., 2024). However, many existing workplace monitoring solutions require high data availability, which is not always feasible in dynamic job roles such as nursing. These findings suggest practical applications for occupational health programs. Complementing this work, investigations into EEG-based brain-computer interfaces for stress detection presented an innovative approach that combines neural indicators with physiological data for comprehensive stress assessment (Premchand et al., 2024). Despite their novelty, EEG-based systems are often intrusive and less practical for long-term stress tracking in daily occupational settings. Real-time prediction models designed for integrating wearable devices into daily life further highlight the practical aspects of these systems (Lazarou and Exarchos, 2024). Nevertheless, most real-time models struggle with handling missing or intermittently collected data, a crucial issue in professional environments.

Recent research has increasingly focused on personalization in stress monitoring solutions. Extensive investigations into wearable-based stress detection in semi-controlled settings identified both opportunities and limitations of current technology (Saini and Gupta, 2024). However, achieving a balance between generalization and personalization remains a challenge in real-world applications. Furthermore, studies proposed generalizable machine learning approaches addressing feature extraction and model generalization across various contexts, enhancing the versatility of stress monitoring systems (Vos et al., 2023). Yet, many approaches still struggle with effectively integrating frequency-domain features, which are essential for capturing stress-related signal variations. Additional research focused on leveraging biosignals for personalized stress detection, demonstrating the efficacy of individual physiological patterns for enhancing predictive accuracy (Bolpagni et al., 2024). However, ensuring model adaptability across different individuals and work environments remains an open problem. Recent developments in real-time physiological data analysis have further advanced personalized stress detection models, facilitating both immediate interventions and longitudinal stress tracking (Ceren Ates et al., 2024). Despite these advances, a unified framework that effectively integrates multi-modal signals for stress detection under real-world intermittent data conditions is still lacking.

Despite these advancements, there remains a need for approaches that effectively integrate both time and frequency domain features from multiple physiological signals within a unified deep learning framework, particularly in the context of intermittent data collection in occupational settings. The current study aims to address this gap by developing a novel multi-modal approach that leverages the strengths of both time and frequency domains for more accurate and robust stress detection. The proposed MMFD-SD model is designed to be flexible and generalizable, capable of handling the challenges of intermittent data collection and varying stress manifestations across different contexts. Utilizing a dataset collected from nurses demonstrates the model’s effectiveness in a high-stress environment; however, the underlying principles and architecture are designed to be applicable across a wide range of occupational and everyday settings.

## 3 Methodology

### 3.1 Overview of the proposed approach

The proposed approach for stress detection leverages a multi-modal deep learning framework that integrates both time and frequency domain features extracted from various physiological signals. The system processes four types of physiological data: accelerometer data (X, Y, Z), EDA, HR, and TEMP. The overall process can be broken down into several key stages: data preprocessing, feature extraction, and deep learning-based classification.

An essential component of this methodology is its acknowledgment of the sporadic nature of wearable sensor data acquisition in occupational environments. This trait is prevalent across multiple industries in the examination of work-related stress, as data is generally gathered during working hours, excluding off-hours or rest periods. Constant 24/7 surveillance is frequently unfeasible or superfluous. The approach is designed to accommodate this intermittent data collection pattern, making it adaptable and applicable to a wide range of occupational stress studies.

In the preprocessing stage, raw physiological signals are cleaned and normalized to remove noise, ensuring data quality for subsequent analysis. Following this, the approach employs a dual-stream feature extraction process. In one stream, a comprehensive set of time-domain features is extracted, including statistical measures and physiological-specific indicators. Concurrently, FFT is applied to the signals, deriving frequency-domain representations from which spectral features are extracted.

The core of this method lies in a custom-designed deep learning architecture that effectively handles this multi-modal data. The architecture consists of two parallel CNN branches: one dedicated to processing time-domain features, and another for frequency-domain features. Each branch is tailored to capture the unique characteristics of its respective domain.

The time-domain CNN branch is designed to learn temporal patterns and relationships within the physiological signals. Similarly, the frequency-domain CNN branch is optimized to identify spectral patterns that may be indicative of stress states. The outputs from these parallel CNN branches are then concatenated, creating a unified representation that encapsulates both temporal and spectral aspects of the physiological data.

This combined representation is then fed into fully connected layers, which perform the final stress classification. By leveraging both time and frequency domain information, the model aims to capture a more comprehensive view of the stress response, potentially leading to more accurate and robust stress detection.

This approach addresses several key challenges in physiological stress detection. By incorporating both time and frequency domain features, it captures a more complete representation of the stress response. The use of parallel CNN branches allows for specialized processing of different feature types, while the subsequent fusion enables the model to leverage complementary information from both domains. Furthermore, by considering the practical constraints of data collection in work environments and designing an algorithm that can effectively process such data, this approach offers a robust and widely applicable solution for stress detection. This adaptability enhances the potential for the method to be used in diverse occupational settings, contributing to broader applications in workplace wellness and stress management.

### 3.2 Data preprocessing

#### 3.2.1 Data segmentation

To handle the discontinuous nature of workplace data collection, a time-based segmentation algorithm is implemented. This algorithm identifies distinct work sessions within the continuous stream of data by analyzing the time intervals between consecutive data points. Let $t_{i}$ represent the timestamp of the *i*th data point. A new segment is defined when the time difference between two consecutive points exceeds a predetermined threshold δ :

$$∆t=t_{i+1}-t_{i}\;>\;\delta$$

where ∆t is the time difference, and δ is set to 900 s (15 min) to account for short breaks or interruptions in data collection.

#### 3.2.2 Data augmentation

To address potential class imbalance and increase the robustness of the model, two data augmentation techniques are employed:

a) Sliding Window: Overlapping segments are generated using a sliding window approach (Gaur et al., 2021; Hou et al., 2022). For a window of size w and step size s, new segments $S_{i}$ are created:

$$S_{i}=\left\{x_{j},x_{j}+1,...,x_{j+w-1}\right\}\text{ for }j=1,1+s,1+2s,...,n-w+1$$

where $x_{j}$ represents the *j*th data point in the original segment.

b) Jittering: Gaussian noise is added to the original data to create slightly perturbed versions (Borghi et al., 2021):

$$x_{i}^{\prime }=x_{i}+\varepsilon$$

where $\varepsilon \;∼\;N\left(0,\sigma^{2}\right)$ , $x_{i}$ is the original data point, $x_{i}^{\prime }$ is the jittered data point, and ε is drawn from a Gaussian distribution with mean 0 and variance $\sigma^{2}$ .

#### 3.2.3 Feature extraction

Two types of features are extracted from each data segment:

a) Time-domain Features: For each physiological signal (X, Y, Z accelerometer axes, EDA, HR, TEMP), statistical measures are computed:

Mean:

$$\mu =\left(1/n\right)\sum x_{i}$$

Standard Deviation:

$$\sigma =\sqrt{\;\left(1/n\right)\sum {\left(x_{i}-\mu \right)}^{2}}$$

b) Frequency-domain Features: FFT is applied to each signal:

$$X\left(k\right)=\sum x\left(n\right)*e^{\left(-j2\pi \text{kn}/N\right)}$$

where k = 0, N-1, $x\left(n\right)$ is the time-domain signal and $X\left(k\right)$ is its frequency-domain representation.

#### 3.2.4 Feature scaling

To ensure all features are on a comparable scale, standardization is applied:

$$z=\left(x-\mu \right)/\sigma$$

where x is the original feature value, μ is the mean of the feature, and σ is its standard deviation.

#### 3.2.5 Class imbalance handling

To address potential class imbalance, the SMOTE is employed (Wang et al., 2021; Wongvorachan et al., 2023). SMOTE generates synthetic examples in the feature space:

$$x_{\text{new}}=x_{i}+\lambda *\left(x_{\text{zi}}-x_{i}\right)$$

where $x_{i}$ is the feature vector under consideration, $x_{\text{zi}}$ is one of its k-nearest neighbors, and $\lambda \in [$ 0,1) is a random number.

This comprehensive preprocessing approach ensures that the subsequent stress detection model is trained on a rich, balanced, and representative dataset. By segmenting the data, augmenting it with realistic variations, extracting both time and frequency domain features, and addressing class imbalance, a robust foundation for accurate stress detection in occupational settings is established.

### 3.3 MMFD-SD architecture

**FIGURE 1:**
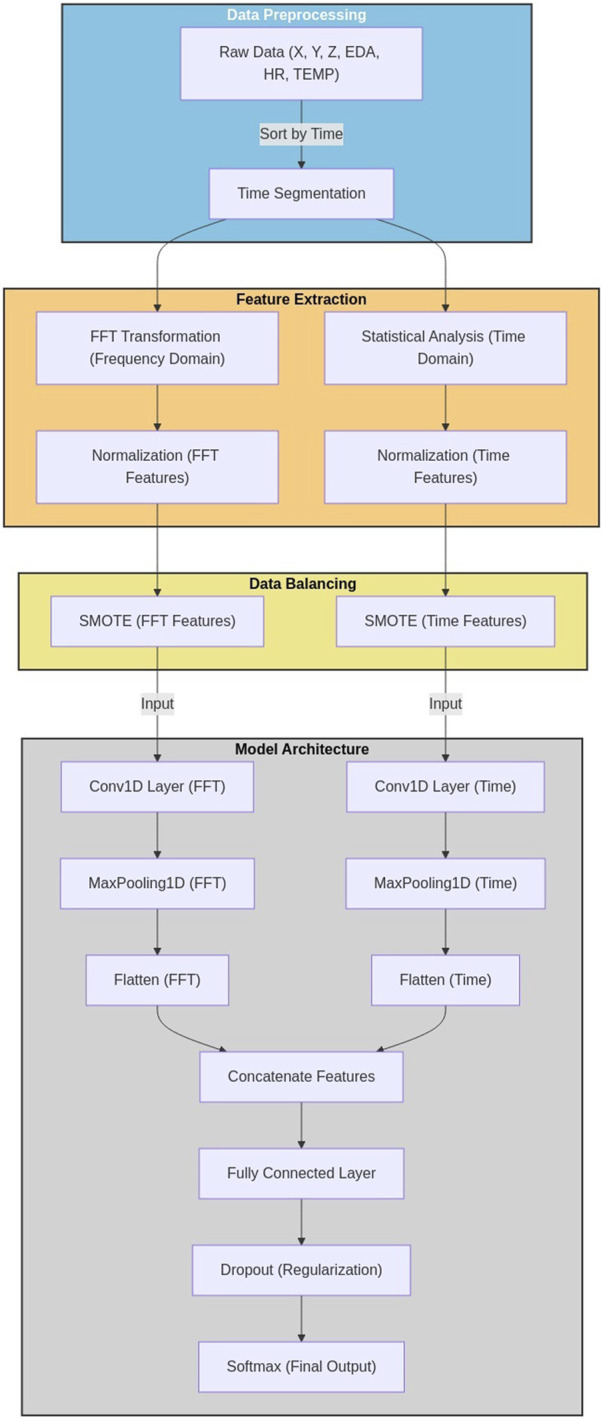
Overall architecture of the MMFD-SD.

The proposed multi-modal deep learning architecture for stress detection leverages both time-domain and frequency-domain features extracted from physiological signals. The architecture consists of three main components: a time-domain CNN branch, a frequency-domain CNN branch, and a feature fusion and classification module. Figure 1 illustrates the overall structure of the proposed model.

The architecture is designed to process input tensors X_t∈R^B×T×F_t for time-domain features and X_f∈R^B×F×F_f for frequency-domain features, where B is the batch size, T is the number of time steps, F is the number of frequency points, and F_t and F_f are the number of time-domain and frequency-domain features, respectively.

#### 3.3.1 CNN for time-domain features

The time-domain CNN branch is designed to capture temporal patterns and local dependencies in the physiological signals. It consists of a series of 1D convolutional layers, each followed by batch normalization, ReLU activation, and max pooling operations.

The l-th convolutional layer can be described by the following equation:

$$H\_l=\mathrm{Pool}\left(\mathrm{ReLU}\left(\mathrm{BN}\left(Conv1D\left(H\_\left\{l-1\right\};\;W\_l,b\_l\right)\right)\right)\right)$$

where H_l is the output of the l-th layer, Conv1D() is the 1D convolution operation, BN() is batch normalization, ReLU() is the rectified linear unit activation function, and Pool () is the max pooling operation. W_l and b_l are the weights and biases of the l-th convolutional layer, respectively.

The final layer of this branch employs global average pooling to produce a fixed-size feature vector:

$$z\_t=\mathrm{GAP}\left(H\_L\right)$$

where GAP() denotes the global average pooling operation, and L is the index of the final convolutional layer.

#### 3.3.2 CNN for frequency-domain features

The frequency-domain CNN branch is structured similarly to the time-domain branch but is optimized for processing spectral information. It operates on the frequency-domain representations of the physiological signals, capturing spectral patterns and frequency-based characteristics.

The mathematical formulation for this branch is analogous to the time-domain branch, with the input being the frequency-domain features X_f instead of X_t.

#### 3.3.3 Feature fusion and classification layers

The feature fusion and classification module combines the outputs from both CNN branches and performs the final classification. This module can be described by the following equations:

$$z=\mathrm{concat}\left(z\_t,z\_f\right)$$

$$h\_1=\mathrm{ReLU}\left(W\_1z+b\_1\right)$$

$$h\_2=\mathrm{ReLU}\left(W\_2h\_1+b\_2\right)$$

$$y=\mathrm{softmax}\left(W\_3h\_2+b\_3\right)$$

where:

concat (z_t, z_f) denotes the concatenation of the time-domain feature vector z_t and the frequency-domain feature vector z_f along the feature dimension. If z_t∈R^d1 and z_f∈R^d2 , then z∈R^d1+d2 .

W_1∈R^m×d1+d2 , W_2∈R^n×m , W_3∈R^K×n are the weight matrices of the fully connected layers.

b_1∈R^m,b_2∈R^n,b_3∈R^K are the corresponding bias vectors

K is the number of classes.

The key innovation in this architecture lies in its ability to simultaneously process and integrate information from both time and frequency domains. This multi-modal approach allows the model to capture a more comprehensive representation of the physiological signals, leading to improved stress detection accuracy.

The parallel CNN branches are designed to extract complementary features: the time-domain branch captures temporal dynamics and trends, while the frequency-domain branch identifies spectral characteristics that may be indicative of stress responses. The subsequent feature fusion enables the model to leverage these complementary representations, allowing for a more nuanced understanding of the complex physiological manifestations of stress.

Furthermore, the use of batch normalization and dropout in both CNN branches helps to stabilize training and prevent overfitting, which is crucial when dealing with the high variability often present in physiological data collected in real-world occupational settings.

The global average pooling layers serve a dual purpose: they reduce the spatial dimensions of the feature maps to a fixed size, regardless of the input dimensions, and they act as a form of structural regularization, encouraging the convolutional filters to produce more informative feature maps.

In summary, this multi-modal deep learning architecture represents a sophisticated approach to stress detection, leveraging advanced deep learning techniques to process and integrate complex physiological data. By combining time-domain and frequency-domain analyses within a unified framework, the model is well-equipped to capture the multifaceted nature of stress responses in occupational environments.

#### 3.3.4 Model training

The training process of the MMFD-SD architecture is crucial for achieving optimal performance in stress detection. A comprehensive approach to model training is employed, carefully considering the loss function, optimization algorithm, hyperparameter tuning, and regularization techniques.

#### 3.3.5 Hyperparameter tuning

Bayesian optimization is employed for hyperparameter tuning, which models the hyperparameter-to-metric function and attempts to find its optimum (Alibrahim and Ludwig, 2021; Victoria and Maragatham, 2020). The acquisition function used in the Bayesian optimization is the Expected Improvement (EI):

$$\mathrm{EI}\left(x\right)=E\left[\max\left(f\left(x\right)-f\left(x+\right),0\right)\right]$$

where $f\left(x+\right)$ is the current best observed value, and $f\left(x\right)$ is the surrogate model’s predicted value at x.

By combining these advanced training techniques and carefully tuning the model, robust and generalizable performance in stress detection across various occupational settings can be achieved. The multi-modal nature of the architecture, coupled with these sophisticated training approaches, allows for effective capture of the complex patterns in physiological data associated with stress responses.

## 4 Experiments

### 4.1 Dataset description

The experiments were conducted using the Nurse Stress Prediction Wearable Sensors dataset, derived from the WESAD dataset, which contains physiological measurements collected from 15 nurses during their hospital work shifts in a real-world clinical environment (Hosseini et al., 2022; Hosseini et al., 2021). The dataset encompasses data recorded using the Empatica E4 wristband, a widely used wearable device for physiological monitoring. Stress levels in the dataset are categorized as Low, Medium, or High, allowing for a granular analysis of stress variations in an occupational setting.

The dataset consists of multi-modal physiological signals, including electrodermal activity (EDA), heart rate (HR), skin temperature (TEMP), and accelerometer data (X, Y, Z-axes). The sampling frequencies for these signals vary, with EDA recorded at 4 Hz, HR at 1 Hz, TEMP at 4 Hz, and accelerometer data at 32 Hz. Table 1 below provides a detailed breakdown of the collected signals and their corresponding sampling rates.

**TABLE 1:** Overview of physiological signals and their frequencies.

Signal	Abbreviation	Frequency
Electrodermal activity	EDA	4.0 Hz
Heart Rate	HR	1.0 Hz
Skin temperature	ST	1.0 Hz
Accelerometer	ACC	32 Hz

The dataset used in this study, while robust for occupational stress research, has limitations. First, it consists of data from a relatively small sample of nurses, which may not fully capture the variability in stress responses across different individuals and work conditions. Second, wearable sensor data is subject to noise, missing values, and artifacts due to movement or device positioning, which can impact signal quality. Addressing these constraints in future work by incorporating larger, more diverse datasets and advanced signal preprocessing techniques would enhance model robustness.

### 4.2 Data collection methodology

The physiological data were collected using the Empatica E4 wristband worn on the non-dominant wrist of each participant throughout their work shifts. The stress labels (Low, Medium, High) were assigned based on self-reported stress levels and physiological indicators, validated through prior methodologies established in occupational stress research.

This dataset provides a robust foundation for developing and evaluating the MMFD-SD model in real-world healthcare settings, as it captures the dynamic and high-stress nature of the nursing profession. By integrating detailed demographic information and real-world stress measurements, this dataset ensures model generalizability and enhances the applicability of stress detection frameworks in occupational health monitoring.

### 4.3 Data preprocessing

The data preprocessing stage involved several key steps to enhance data quality and prepare it for model training. The collected physiological signals were first segmented into 60-s windows with 50% overlap to create consistent data samples. Each physiological signal underwent min-max scaling for normalization. Following the preprocessing steps described earlier, the data underwent time-based segmentation using a 15-min threshold to identify distinct work sessions, data augmentation through sliding windows and Gaussian jittering, and feature extraction in both time and frequency domains (Alqudah and Alqudah, 2019; Xie et al., 2020). Finally, the SMOTE technique was applied to address class imbalance, resulting in a balanced and representative dataset suitable for stress detection modeling.

### 4.4 Model design

The proposed MMFD-SD algorithm presents a comprehensive deep learning approach for stress detection using multi-modal physiological signals. The algorithm processes both time-domain and frequency-domain features through parallel CNN branches, each consisting of multiple convolutional layers with batch normalization, ReLU activation, and max pooling operations (Fu et al., 2022). The extracted features are then concatenated and fed into a fusion classifier comprising fully connected layers for final stress level classification. The training process utilizes mini-batch gradient descent with early stopping, incorporating cross-entropy loss and L2 regularization to prevent overfitting. The algorithm’s modular structure allows for efficient processing of different signal modalities while maintaining end-to-end training capabilities. The detailed implementation of the algorithm is presented in Algorithm 1 below.

Algorithm 1 MMFD-SD.

**Input**: Time-domain features X_t, Frequency-domain features X_f, Labels y,
  Hyperparameters η (learning rate), λ (L2 regularization coefficient),
  Number of epochs E, Batch size B
**Output**: Trained model parameters Θ

1: Initialize model parameters $\Theta =\{\Theta \_t,\Theta \_f,\Theta \left.\_\text{fc}\right\}$ randomly
2: **for** each epoch e = 1, 2, E **do**
3:  Shuffle training data
4:  **for** each batch b of size B **do**
5:   // Forward pass
6:   z_t ← TimeDomainCNN(X_t; Θ _t)
7:   z_f ← FrequencyDomainCNN(X_f; Θ_f )
8: $z\;\leftarrow \text{ Concat}\left(z\_t,z\_f\right)$

9: $y\_\text{pred }\leftarrow \text{ FusionClassifier}\left(z;\;\Theta \_\text{fc}\right)$

10:   // Compute loss
11: $L\;\leftarrow \text{CrossEntropyLoss}\left(y\_\text{pred},y\right)$ $+\lambda \;*\;L2\text{Regularization}\left(\Theta \right)$

12:   // Backward pass
13: $\nabla \Theta \leftarrow \text{Backpropagate}\left(L,\Theta \right)$

14:   // Update parameters
15: Θ←Θ−η*∇Θ Θ
16:  **end for**
17:  // Validation
18:  **if** EarlyStoppingCriterionMet () **then**
19:   break
20:  **end if**
21: **end for**
22: **return** Θ

**Function** $\text{TimeDomainCNN}\left(X\_t;\Theta \_t\right)$

1: **for** each convolutional layer l **do**
2:  X_t ← Conv1D (X_t)
3:  X_t ← BatchNorm (X_t)
4:  X_t ← ReLU(X_t)
5:  X_t ← MaxPool (X_t)
6: **end for**
7: z_t ← GlobalAveragePooling (X_t)
8: **return** z_t
**Function** FrequencyDomainCNN(X_f; Θ_f )
1: **for** each convolutional layer l **do**
2:  X_f ← Conv1D (X_f)
3:  X_f ← BatchNorm (X_f)
4:  X_f ← ReLU(X_f)
5:  X_f ← MaxPool (X_f)
6: **end for**
7: z_f ← GlobalAveragePooling (X_f)
8: **return** z_f
**Function** FusionClassifier(z; Θ_f c)
1: h_1 ← ReLU(FullyConnected(z))
2: h_2 ← ReLU(FullyConnected (h_1))
3: y_pred ← Softmax (FullyConnected (h_2))
4: **return** y_pred

### 4.5 Evaluation metrics

The model’s performance was evaluated using the following metrics:

$$\mathrm{Accuracy}=\left(\mathrm{TP}+\mathrm{TN}\right)\;/\;\left(\mathrm{TP}+\mathrm{TN}+\mathrm{FP}+\mathrm{FN}\right)$$

$$\mathrm{Precision}=\mathrm{TP}/\;\left(\mathrm{TP}+\mathrm{FP}\right)$$

$$\mathrm{Recall}=\mathrm{TP}/\;\left(\mathrm{TP}+\mathrm{FN}\right)$$

$$F1-\mathrm{score}=2\;*\;\left(\mathrm{Precision}\cdot \mathrm{Recall}\right)\;/\;\left(\mathrm{Precision}+\mathrm{Recall}\right)$$

where TP = True Positives, TN = True Negatives, FP = False Positives, FN = False Negatives.

## 5 Results

This section provides a comprehensive analysis of the MMFD-SD’s performance, including baseline experiments, ablation studies, and sensitivity analyses. Baseline experiments establish the model’s accuracy and robustness by comparing it with various stress detection algorithms, including traditional machine learning classifiers. Ablation studies offer insight into the contribution of individual components, such as the time-domain and frequency-domain branches, by incrementally removing modules. Sensitivity analysis focuses on evaluating the effect of hyperparameters on model performance, aiming to determine optimal settings while validating the model’s stability and adaptability across different configurations.

### 5.1 Baselines and MMFD-SD

Several baseline models, including Logistic Regression, Naive Bayes, Random Forest, Decision Tree, K-Nearest Neighbors (KNeighbors), AdaBoost, and XGBoost was conducted. These models were selected due to their prevalent use and effectiveness in various classification tasks.

The performance of each model was assessed using multiple metrics: accuracy, precision, recall, and F1-score. The results are compared to the performance of the MMFD-SD model, which serves as a benchmark for evaluating the effectiveness of the baseline approaches. The detailed results are summarized in the Table 2 below.

**TABLE 2:** Performance metrics of different models for stress detection.

Model	Accuracy	Precision	Recall	F1-score
LogisticRegression	0.7419	0.5564	0.7419	0.6321
NaiveBayes	0.7374	0.6565	0.7374	0.6415
RandomForest	0.6336	0.3976	0.3898	0.3918
Decision Tree	0.57	0.3733	0.3791	0.3718
KNeighbors	0.5901	0.3395	0.3402	0.3396
AdaBoost	0.6851	0.3616	0.3488	0.3427
XGBoost	0.6483	0.4087	0.3964	0.4003
MMFD-SD	0.91	0.91	0.91	0.91
LogisticRegression	0.7419	0.5564	0.7419	0.6321

Logistic Regression achieved an accuracy of 74.19%, demonstrating its capability for linear decision boundaries. Naive Bayes performed similarly, with an accuracy of 73.74%, reflecting its efficiency in handling categorical data and independence assumptions.

Random Forest yielded a lower accuracy of 63.36%, indicating that the model may not have effectively captured the underlying patterns in this dataset. In contrast, the Decision Tree model displayed an accuracy of 57.00%, suggesting that its tendency to overfit may have impacted its generalizability.

The K-Nearest Neighbors classifier achieved an accuracy of 59.01%, while AdaBoost showed improved performance with an accuracy of 68.51%. XGBoost, known for its scalability and performance, obtained an accuracy of 64.83%.

In stark contrast, the proposed model, MMFD-SD, significantly outperformed all baseline models, achieving an accuracy of 91.00%. This substantial improvement underscores the effectiveness of the MMFD-SD approach in achieving higher predictive performance.

**FIGURE 2:**
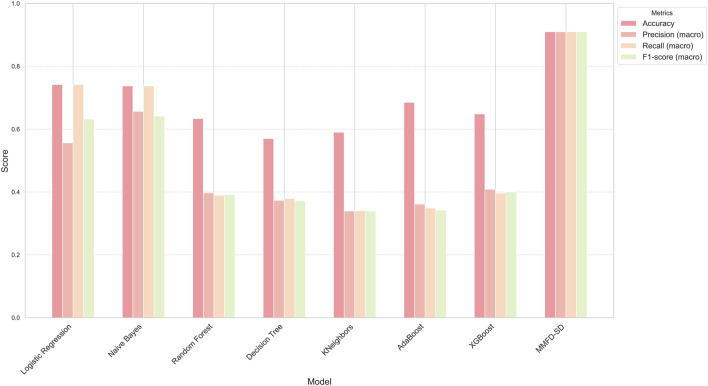
Performance comparison of baseline models and MMFD-SD.

The performance metrics are illustrated in the accompanying bar chart, which visually represents the comparative analysis of each model’s accuracy, precision, recall, and F1-score. The chart distinctly separates the metrics, allowing for a clear interpretation of each model’s strengths and weaknesses. The MMFD-SD model stands out prominently across all metrics, achieving the highest scores and highlighting its superiority compared to the baseline models. The overall trends depicted in the chart indicate the varying effectiveness of the baseline models, with MMFD-SD significantly surpassing them in all evaluated aspects. The results are summarized in the following Figure 2.

### 5.2 Ablation studies

In the ablation studies, the impact of individual feature domains on the performance of the MMFD-SD is analyzed by evaluating two variant models: the No-Freq Model, which excludes frequency-domain features, and the No-Time Model, which excludes time-domain features. These variations are compared against the original model, which integrates both feature domains. The results of this comparison provide insights into the contribution of each feature type, demonstrating how each domain affects overall classification accuracy, precision, recall, and F1-score in stress detection. This analysis aims to determine the relative importance of each feature set and validate the advantages of multimodal feature integration in the MMFD-SD model.

#### 5.2.1 Classification report for No-Freq model

The classification report for the No-Freq Model indicates its performance in detecting stress levels without utilizing frequency-domain features. The precision, recall, and F1-score metrics for each stress level demonstrate that this model achieves a precision of 0.79 for Class 0, 0.85 for Class 1, and 0.84 for Class 2, with a notable recall of 0.87 for Class 0 but a lower recall of 0.70 for Class 2. The overall accuracy of the model stands at 0.83, reflecting its capability to classify stress levels adequately, albeit with some limitations, especially for Class 2. The macro averages are 0.83 for precision and recall, and 0.82 for F1-score, suggesting a relatively balanced performance, with potential areas for improvement in classification accuracy for Class 2 as shown in Table 3.

**TABLE 3:** Classification performance metrics for No-Freq model across stress levels.

Class	Precision	Recall	F1-score
0	0.79	0.87	0.83
1	0.85	0.91	0.88
2	0.84	0.7	0.77
Macro Avg	0.83	0.83	0.82
Accuracy	0.83		

#### 5.2.2 Classification report for No-Time model

The classification report for the No-Time Model provides an evaluation of the model’s performance when excluding time-domain features. This model exhibits improved precision and recall metrics compared to the No-Freq Model, with precision scores of 0.86 for Class 0, 0.88 for Class 1, and 0.89 for Class 2. The recall rates also show significant improvement, reaching 0.88 for Class 0 and 0.96 for Class 1, with a slightly lower recall of 0.80 for Class 2. The overall accuracy of 0.88 indicates that the model effectively classifies stress levels, particularly excelling in distinguishing between Classes 0 and 1. The macro averages of 0.88 across precision, recall, and F1-score confirm the robustness of this model, highlighting its effectiveness in stress detection despite the absence of time-domain features as shown in Table 4.

**TABLE 4:** Classification performance metrics for No-time model across stress levels.

Class	Precision	Recall	F1-score
0	0.86	0.88	0.87
1	0.88	0.96	0.92
2	0.89	0.80	0.84
Macro Avg	0.88	0.88	0.88
Accuracy	0.88		

#### 5.2.3 Classification report for MMFD-SD model

The classification report for the MMFD-SD Model illustrates its superior performance in stress detection by integrating both time and frequency-domain features. The precision scores are notably high, with 0.89 for Class 0, 0.94 for Class 1, and 0.91 for Class 2, indicating effective classification across all stress levels. The recall metrics also reflect strong performance, achieving 0.92 for Class 0, 0.95 for Class 1, and 0.87 for Class 2, resulting in a balanced F1-score of 0.91 for each class. The overall accuracy of the model is 0.91, underscoring its capability to accurately classify stress levels. The macro averages of 0.91 across precision, recall, and F1-score highlight the effectiveness of the MMFD-SD model, confirming its robustness and adaptability in stress detection tasks as shown in Table 5.

**TABLE 5:** Classification performance metrics for MMFD-SD model across stress levels.

Class	Precision	Recall	F1-score
0	0.89	0.92	0.91
1	0.94	0.95	0.95
2	0.91	0.87	0.89
Macro Avg	0.91	0.91	0.91
Accuracy	0.91		

#### 5.2.4 Comparison of Confusion Matrices for Different Models

**FIGURE 3:**
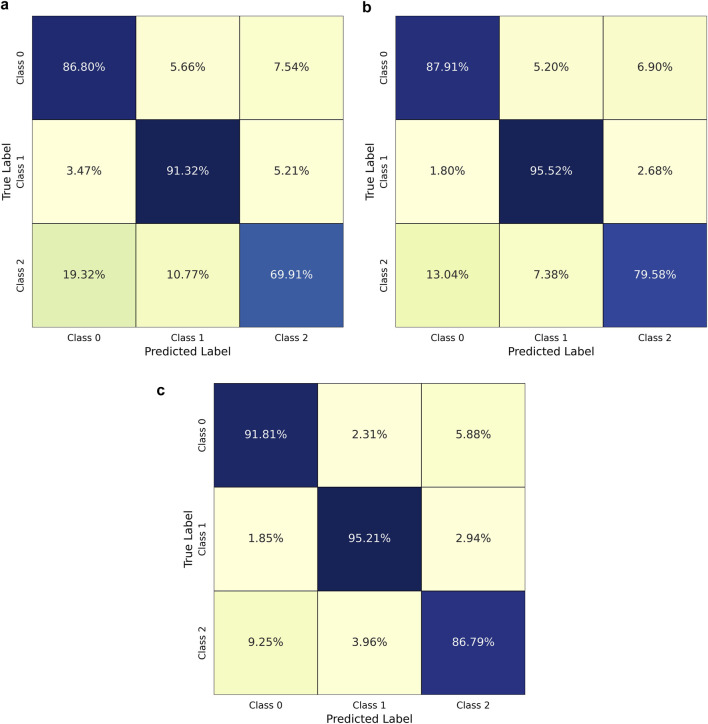
Comparison of confusion matrices for different models. (**a**) No-Freq Model - Confusion Matrix; (**b**) No-Time Model - Confusion Matrix; (**c**) MMFD-SD Model - Confusion Matrix.

The confusion matrices displayed in the accompanying Figure 3 illustrate the classification performance of three models: the MMFD-SD Model, the No-Freq Model, and the No-Time Model. Each matrix presents the percentage of predictions across three stress levels (Class 0, Class 1, and Class 2).

The MMFD-SD Model demonstrates superior performance, with high true positive rates for all classes, indicating effective differentiation between stress levels. In contrast, the No-Freq Model shows a decline in classification accuracy, particularly for Class 2, where misclassifications are more prevalent. Similarly, the No-Time Model reveals further limitations, with a notable increase in false positive rates across all classes.

Overall, the visual comparison underscores the importance of integrating both time and frequency-domain features in achieving optimal stress detection performance. The percentages reflect how well each model can classify the stress levels, emphasizing the advantages of the multimodal approach employed by the MMFD-SD Model.

**FIGURE 4:**
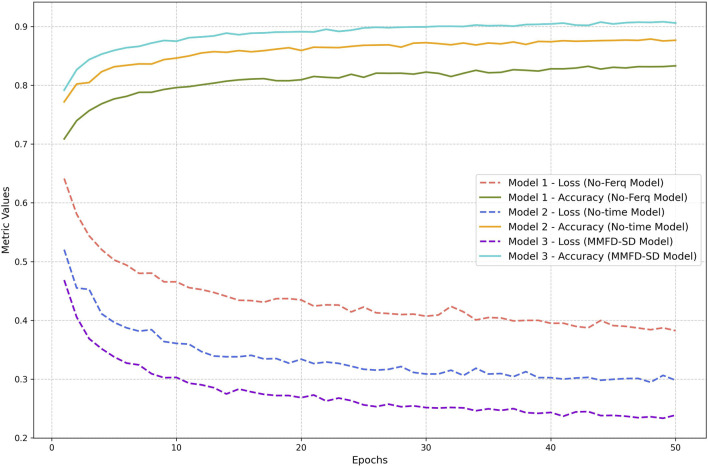
Validation metrics comparison.

Figure 4 presents the validation loss and accuracy across 50 training epochs for three models: the No-Ferq Model, the No-time Model, and the MMFD-SD Model.

When only time-domain features were utilized, the model exhibited moderate performance. The validation accuracy plateaued at 83%, with the loss showing gradual improvement but remaining higher than other models.

The No-time Model solely employed frequency-domain features, showed better performance compared to the No-Ferq Model. The validation accuracy reached around 88%, and the loss consistently declined, reflecting improved generalization.

The MMFD-SD Model integrating both time- and frequency-domain features, this model achieved the best results. It consistently outperformed the other two models in validation accuracy, surpassing 90%, while maintaining the lowest validation loss across all epochs.

The results highlight the critical role of feature integration in enhancing model performance. The MMFD-SD Model, leveraging both feature domains, demonstrates superior accuracy and stability, affirming the effectiveness of a multi-modal approach for stress detection.

### 5.3 Sensitivity analysis of hyperparameters

The sensitivity analysis of hyperparameters was conducted to evaluate the impact of varying learning rates, dropout rates, and batch sizes on the model’s performance. The experiments involved combinations of three learning rates (0.001, 0.0001, and 1e-05), three dropout rates (0.3, 0.5, and 0.7), and three batch sizes (32, 64, and 128). To ensure a comprehensive assessment, each configuration was trained for a fixed number of epochs (50 epochs) using the Adam optimizer, which is known for its adaptive learning rate capabilities. The selection of these hyperparameters was based on prior research in deep learning-based physiological signal processing, ensuring relevance to stress detection tasks. For the optimization process, a grid search approach was employed to systematically evaluate all possible combinations of the selected hyperparameters. Each model configuration was trained on 80% of the dataset and validated on the remaining 20% using a stratified split to maintain the distribution of stress levels.

The results, summarized in the following tables, indicate that the combination of a learning rate of 0.001, a dropout rate of 0.3, and a batch size of 64 yielded the highest test accuracy of 0.9062 as shown in table 6.

**TABLE 6:** Impact of different learning rates, dropout rates, and batch sizes on model test accuracy.

Batch size	Learning rate	Dropout rate	Test accuracy
32	0.001	0.3	0.9006
	0.0001	0.3	0.888
	1e-05	0.3	0.8219
	0.001	0.5	0.8833
	0.0001	0.5	0.8613
	1e-05	0.5	0.8041
	0.001	0.7	0.8439
	0.0001	0.7	0.8382
	1e-05	0.7	0.7768
64	0.001	0.3	0.9062
	0.0001	0.3	0.8825
	1e-05	0.3	0.8038
	0.001	0.5	0.8878
	0.0001	0.5	0.8672
	1e-05	0.5	0.7875
	0.001	0.7	0.8501
	0.0001	0.7	0.8131
	1e-05	0.7	0.7552
128	0.001	0.3	0.9037
	0.0001	0.3	0.8771
	1e-05	0.3	0.7896
	0.001	0.5	0.8934
	0.0001	0.5	0.8548
	1e-05	0.5	0.7529
	0.001	0.7	0.856
	0.0001	0.7	0.821
	1e-05	0.7	0.7341

To provide a more comprehensive view of the results, various visualizations were generated.

#### 5.3.1 Test accuracy by dropout rate

**FIGURE 5:**
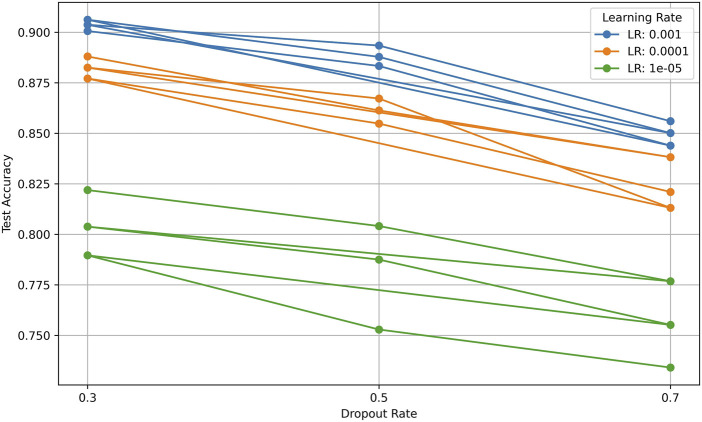
Test accuracy by dropout rate.

The line chart illustrates how test accuracy varies with dropout rates for different learning rates. It is evident that a dropout rate of 0.3 consistently results in higher accuracy across all learning rates, with the 0.001 learning rate achieving the best performance as shown in Figure 5. This suggests that a lower dropout rate may help retain more important features, leading to better generalization.

#### 5.3.2 Heatmap of Test Accuracy

**FIGURE 6:**
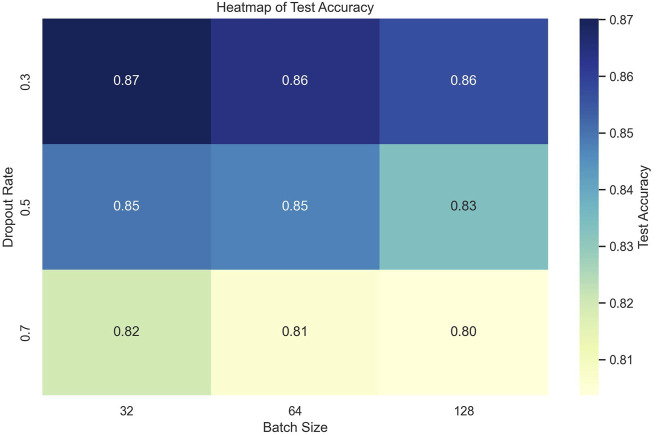
Heatmap of test accuracy.

The heatmap provides a clear visualization of the test accuracy across different combinations of dropout rates and batch sizes. Each cell represents the accuracy achieved for a specific combination, with darker shades indicating higher accuracy as shown in Figure 6. The optimal performance is observed with a batch size of 64 and a dropout rate of 0.3, reaffirming the results from the earlier analysis.

#### 5.3.3 Test accuracy by batch size

**FIGURE 7:**
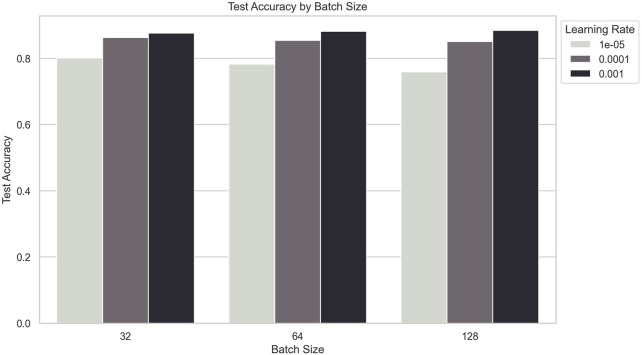
Test accuracy by batch size.

The bar plot compares the test accuracy across different batch sizes while differentiating between learning rates. The highest accuracy is achieved with a batch size of 64, particularly at a learning rate of 0.001 as shown in Figure 7. This indicates that both the choice of batch size and learning rate significantly influence model performance, highlighting the importance of careful hyperparameter tuning.

## 6 Discussion

This study presents MMFD-SD, a novel multi-modal deep learning framework specifically designed for stress detection using multiple physiological signals. By integrating both time-domain and frequency-domain features from accelerometer data, EDA, HR, and TEMP, MMFD-SD captures a holistic representation of the stress response that surpasses traditional single-domain or single-modal methods. The inclusion of FFT-based spectral features complements the time-domain features, providing a more comprehensive view of the stress response by capturing valuable information that may be overlooked when relying solely on time-domain analysis. This integration of multi-domain features has been demonstrated to significantly enhance classification performance, as reflected in our experimental results.

Additionally, the custom architecture—utilizing parallel CNNs to separately process time and frequency domains—enables effective multi-modal feature extraction and enhances classification accuracy by capturing complex patterns across domains. Compared to traditional machine learning classifiers such as Support Vector Machines (SVM) and Random Forest (RF), our approach achieves superior stress detection accuracy. Experimental results show that MMFD-SD outperforms these conventional models, highlighting the effectiveness of deep learning in physiological signal processing.

Furthermore, MMFD-SD is designed to address the unique challenges of occupational stress monitoring, particularly the intermittent nature of data collection constrained to work hours. This aspect is critical, as existing stress detection models often assume continuous monitoring, which may not be practical in workplace settings. This adaptation acknowledges the real-world limitations of wearable sensor data in professional settings, focusing analysis on periods most relevant to occupational stress. Our findings suggest that intermittent data collection does not significantly degrade model performance, reinforcing the feasibility of MMFD-SD for real-world deployment.

To further validate the model’s effectiveness, we conducted extensive ablation studies and sensitivity analyses. The ablation study confirmed that the inclusion of both time-domain and frequency-domain features led to a noticeable improvement in classification accuracy, reinforcing the importance of multi-domain feature fusion. Sensitivity analysis indicated that a learning rate of 0.001, a dropout rate of 0.3, and a batch size of 64 provided optimal performance, balancing convergence speed and generalization capability.

While this study focuses on stress detection in nurses, the proposed MMFD-SD model is designed to generalize to other occupational settings where stress monitoring is critical. High-stress professions such as emergency responders, pilots, and industrial workers share similar physiological responses to stress. The intermittent data collection framework ensures adaptability to work environments where continuous monitoring is impractical. Future research could validate the model’s effectiveness in different workplaces by collecting and analyzing datasets from diverse occupational groups.

Despite its strong performance, MMFD-SD has some limitations. The model currently relies on supervised learning, which requires labeled training data. Future work could explore semi-supervised or self-supervised approaches to mitigate data labeling constraints. Additionally, integrating contextual information, such as work shift duration and task intensity, could further enhance stress prediction accuracy.

Overall, the results demonstrate that MMFD-SD offers a highly effective approach for stress detection using intermittently collected wearable sensor data. By addressing the limitations of previous methods and leveraging both time and frequency-domain features, this study contributes valuable insights into affective computing and occupational stress monitoring. These findings suggest potential applications not only in healthcare worker wellness but also in broader fields such as mental health monitoring and personalized stress management.

## 7 Conclusion

The proposed MMFD-SD model demonstrates substantial advancements in stress detection compared to baseline models. By effectively integrating time-domain and frequency-domain features, MMFD-SD provides superior performance, achieving the highest accuracy and robustness across multiple evaluation metrics. Ablation studies confirm that each feature domain contributes significantly to the model’s success, supporting the effectiveness of the multimodal approach.

Sensitivity analysis further validated the model’s adaptability, identifying optimal hyperparameter settings that balance performance with stability. This adaptability, coupled with the robustness seen in baseline comparisons, highlights MMFD-SD’s suitability for diverse stress detection tasks.

Future research could explore additional feature domains or employ alternative integration techniques to further enhance performance, particularly in highly variable stress datasets, as well as the integration of real-time stress detection capabilities and testing across broader datasets to improve its generalizability and real-world applicability. Overall, the MMFD-SD model stands as a reliable and advanced solution for real-world stress detection applications.

## Data Availability

Publicly available datasets were analyzed in this study. This data can be found here: https://datadryad.org/stash/dataset/doi:10.5061/dryad.5hqbzkh6f#citations.
